# Old Plant, New Possibilities: Wild Bilberry (*Vaccinium myrtillus* L., Ericaceae) in Topical Skin Preparation

**DOI:** 10.3390/antiox10030465

**Published:** 2021-03-16

**Authors:** Vanja M. Tadić, Ivana Nešić, Milica Martinović, Edward Rój, Snežana Brašanac-Vukanović, Svetolik Maksimović, Ana Žugić

**Affiliations:** 1 Department for Pharmaceutical Research and Development, Institute for Medicinal Plant Research “Dr. Josif Pancic”, Tadeusa Koscuska 1, 11000 Belgrade, Serbia; 2 Department of Pharmacy, Faculty of Medicine, University of Nis, Boulevard Dr. Zorana Djindjića 81, 18000 Nis, Serbia; ivana.nesic@medfak.ni.ac.rs (I.N.); milica.martinovic@medfak.ni.ac.rs (M.M.); 3 Supercritical Extraction Department, Łukasiewicz Research Network—New Chemical Syntheses Institute, Al. Tysiąclecia Państwa Polskiego 13a, 24-110 Puławy, Poland; edward.roj@ins.lukasiewicz.gov.pl; 4 Faculty of Metallurgy and Technology, University of Montenegro, Dzordza Vasingtona bb, 20000 Podgorica, Montenegro; sneza_b@yahoo.com; 5 Department of Organic Chemical Technology, Faculty of Technology and Metallurgy, University of Belgrade, Karnegijeva 4, P.O. Box 3503, 11120 Belgrade, Serbia; smaksimovic@tmf.bg.ac.rs

**Keywords:** *Vaccinium myrtillus-bilberry*, bilberry leaves extract, bilberry seed oil, skin preparation, antioxidant activity, in vivo skin performance

## Abstract

Bilberry represents a valuable source of antioxidant substances responsible for its application for the treatment of different conditions (such as inflammation, cardiovascular disease, cancer, diabetes, and different age-related diseases) associated with increased oxidative stress. As oxidative stress might cause skin impairments, we aim to evaluate a topical preparation containing bilberry leaves extract and bilberry seeds oil, obtained as a byproduct of the food industry. To obtain the extracts, the conventional maceration technique for leaves, and supercritical carbon dioxide extraction for seeds were employed. The chemical profile of both actives was achieved by HPLC and GC methods, revealing the presence of phenolic acids (chlorogenic being the most abundant), flavonoids (isoquercetin in the highest amount), and resveratrol in leaves extract, while in seeds oil the essential ω-3 and ω-6 fatty acids were determined in favorable ratio, almost being 1. Antioxidant potential of the wild bilberry extract and seed oil was evaluated using in vitro DPPH and FRAP assays. Finally, effects of the oil-in-water creams with mentioned wild bilberry isolates on the skin were investigated in an in vivo study conducted on healthy human volunteers, revealing the significant beneficial effects when topically applied.

## 1. Introduction

Wild bilberry (*Vaccinium myrtillus* L.), a member of the Ericaceae family, is a low-growing shrub native to northern Europe and North America. Aside other phenolic compounds, the main phenolic constituent of the berries of this plant is anthocyanins, further connected to their many reported beneficial health effects, such as lowering blood glucose, anti-inflammatory and lipid-lowering effects, as well as promotion of antioxidant defense and reduction of oxidative stress. Hence, bilberry fruits represent attractive food product potentially valuable in the prevention and/or treatment of different conditions associated with inflammation, dyslipidemia, hyperglycemia or increased oxidative stress, cardiovascular disease (CVD), cancer, diabetes, and dementia and other age-related diseases. Aside being sold as fresh, frozen, and dried whole berries, wild bilberry fruits are also often used in the production of preserves, jams, and juices, as well as liquid or powdered concentrates used as food supplements [1,2].

In the processing of wild bilberry fruits, certain waste or by-products may be expected, such as leaves, seeds, and peels that are usually underutilized, despite being a rich source of bioactive compounds [1]. For instance, wild bilberry leaves are reported to be abundant in various phenolic compounds, with flavonoids, phenolic acids, tannins, and stilbenes in higher amounts compared to wild bilberry fruits and consequently, to possess enhanced antioxidant properties [3]. In this connection, they have been used as a traditional remedy in the control of blood sugar [4]. On the other hand, bilberry seeds, which represent around 2.9% of the whole berry, contain around 30.5% of seed oil, rich in polyunsaturated fatty acids (PUFAs) and a high level of antioxidants, such as vitamin E [1]. Although oils are usually extracted from seeds using solvent extraction or cold pressing, these methods have certain disadvantages such as the usage of organic solvents/high temperatures for the former and low yields for the latter. Therefore, in recent years supercritical fluid extraction (SFE) has been used as an alternative method over traditional extraction technologies due to its numerous advantages reflected primarily in a flexible process, allowing production of extracts with desired fractions of chemical constituents, free from polluting organic solvents. One of the most used supercritical fluids is certainly carbon dioxide (CO_2_), as it is inert, inexpensive, easily available, odorless, tasteless, environment-friendly, and GRAS (generally regarded as safe) solvent. In addition, its near-ambient critical temperature (31.1 °C) makes it ideally suitable for thermolabile natural products [5]. 

With respect to the above-mentioned, due to their chemical composition i.e., the presence of phenolic constituents and PUFAs, waste products of bilberry fruits production, i.e., leaves and seeds may be potentially used in food or cosmetic industry. Therefore, in this study, the main objective is to investigate the potential of the residues of food products in the topical skin preparations. Hence, we prepared bilberry leaves extract by means of maceration with ethanol as a commonly used technique for the extraction of polyphenolic compounds [6] and oil prepared from bilberry seed using SFE with CO_2_. Afterwards their chemical composition was assessed using high pressure liquid chromatography (HPLC) and gas chromatography mass spectrometry (GC/MS) methods, while their antioxidant activity, potentially related to their usage in prevention of oxidative-stress related conditions, including skin aging was tested using two in vitro assays (DPPH and FRAP). Finally, stated wild bilberry isolates (leaves macerate and seed oil) were added into oil-in-water (O/W) cream as active compounds and its effects on the skin were investigated in an in vivo study conducted on healthy human volunteers. Furthermore, evaluation of their sensory characteristics, often decisive for the consumer acceptance being the biggest part of the topical products’ sales potential [7] was assessed as well.

## 2. Materials and Methods

### 2.1. Materials

Analytical grade reagents acetate buffer, 2,4,6-tripyridyl-s-triazine (TPTZ), HCl, FeCl_3_, Folin-Ciocalteu reagent,2,6-di-*tert*-butyl-4-methylphenol (BHT), *n*-butanol (BuOH), acetone, ethyl acetate, sodium bicarbonate, 1,10-diphenyl-2-picrylhydrzyl (DPPH), absolute ethanol (96%, *v*/*v*), sodium bicarbonate and methanol were purchased from Sigma-Aldrich (St. Louis, MO, USA). Acetonitrile, water, and methanol (HPLC grade) were purchased from Merck (Darmstadt, Germany). Reference HPLC standards, gallic acid, pyrogallol, protocatechuic acid, chlorogenic acid, procyanidin B2, vanilic acid, caffeic acid, syringic acid, epicatechin, *p*-coumric acid, ferulic acid, sinapic acid, rutin, hyperoside, isoquercetin, kaempferol-3-*O*-glukoside, resveratrol, quercetin, kaempferol (purity *≥* 99%) were purchased from Extrasynthese (Genay, France).

Plant material used in this study were dried herbal parts (bilberry fruits and leaves), collected at Bjelasica Mountain, Montenegro. The voucher specimens (No VMF_121215, and VML_111215) were deposited at the Faculty of Pharmacy, Department of Botany, University of Belgrade, where the identification was performed. Shortly before the extraction, leaves were ground in an electric mill (all samples powdered, dimension of the particles 355, as recommended in the European Pharmacopoeia 9.0 [8]). The seeds were separated from the pulp, washed in water, and dried before extraction.

Myritol^®^318 was purchased from Henkel (Dusseldorf, Germany), while Sabowax^®^AE and glycerin were acquired from Sabo (Levate, Italy). Lanette 16 and stearyl alcohol were bought from Cognis (Monheim, Germany), while sodium benzoate was obtained from Zdravlje (Leskovac, Serbia) and purified water was from Medical faculty in Niš (Serbia).

### 2.2. Preparation of Wild Bilberry Isolates

#### 2.2.1. Preparation of Wild Bilberry Leaves Extract

The extract of wild bilberry leaves was prepared using the method of maceration. Namely, 50 g of the grounded herbal drug was mixed with 300 mL of 70% ethanol and the extraction was carried out at room temperature with occasional stirring. After 72 h, the extract was filtered through filter paper and the precipitate was re-extracted with 20 mL of the fresh solvent. After 48 h, the process was repeated and the combined supernatants were evaporated to dryness. The extraction yield was 16.1%.

#### 2.2.2. Preparation of Wild Bilberry Seeds Oil

Extraction of wild bilberry seeds oil with supercritical CO_2_ was carried out using a laboratory plant produced by SITEC (Maur, Switzerland). The unit consisted of a CO_2_ pump (Lewa company, Leonberg, Germany) connected to a pump head cooling device (Julabo GmbH, Seelbach, Germany), an automatic back pressure regulator to control the pressure, a 600 mL extraction basket surrounded by a heating jacket, and two separation vessels S1 and S2, following operating parameters: S1-pressure up to 500 bar and temperature up to 80 °C and S2-pressure up to 100 bar and temperature up to 50 °C. Triple crushed seeds (100 ± 1 g) were loaded into the extraction basket. The filters at the inlet and outlet of the basket were made of sintered metal plates with a pore size of 7 µm. The extraction was performed at a pressure of 300 bar and a temperature of 40 °C. One separation step was used. The CO_2_ consumption was 60 kg CO_2_/kg at the flow of 5 kg/h. After extraction, the oils were stored at −20 °C until analyzed. The extraction yield was 31.2%.

### 2.3. Chemical Analysis of Wild Bilberry Isolates

#### 2.3.1. Determination of Total Phenolics, Tannins, Flavonoids Content in Wild Bilberry Leaves Extract

All of the following measurements were assessed spectrophotometrically using UV-VIS Spectrophotometer HP 8453 (Agilent Technologies, Santa Clara, CA, USA).

Total phenolic (TP) content was determined by the Folin-Ciocalteu method: one hundred microliter of methanolic solution of the investigated extract (0.49 mg/mL) was mixed with 0.75 mL of Folin-Ciocalteu reagent that was previously diluted ten-fold with distilled water and allowed to stand at 22 °C for 5 min, after which 0.75 mL of sodium bicarbonate (60 g/L) solution was added to the mixture. After 90 min at 22 °C, absorbance of this mixture was measured at λmax 725 nm. Gallic acid (0–100 mg/L) was used for calibration of a standard curve which showed the linear regression at r > 0.99, and the results are expressed as mg of gallic acid equivalents per g of plant extract dry weight (mg GAE/g DW). The content of TP presented the mean of three determinations.

The percentage content of TT was calculated using the method described in the European Pharmacopoeia 9.0 [8]: Decoctions prepared from the investigated extract were treated with phosphomolybdotungstic reagent in alkaline medium with and without treatment with hide powder. The absorbance was measured at λmax 760 nm. The percentage of tannins (the mean of three determinations) was expressed as pyrogallol (%, *w*/*w*) and was calculated from the difference in absorbance of total polyphenols (A_1_) and polyphenols not adsorbed by hide powder (A_2_), using the following expression:

62.5(A_1_ − A_2_) × m_2_/(A_3_ − m_1_) (1)

where m_1_ represented the mass of the sample to be examined, in grams; and m_2_ mass of pyrogallol, in grams.

The content of TF was calculated using the method described in the European Pharmacopoeia 9.0 [8]. Briefly, the sample was extracted with acetone/HCl under reflux condenser; the AlCl_3_ complex of flavonoid fraction extracted by ethyl acetate was measured spectrophometrically at λmax 425 nm. The content of flavonoid (mean of three determinations), was expressed as hyperoside percentage, and was calculated using the following expression:

A × 1.25/m(2)

where A was absorbance at 425 nm and m was the mass of the extracts to be examined in grams.

#### 2.3.2. HPLC Analysis of Wild Bilberry Leaves Extract

“Fingerprinting” of the investigated phenolic compounds was carried out using 1200 HPLC system (Agilent Technologies) equipped with Lichrospher 100RP 18e column, applying gradient elution of two mobile phases, (phase A being 0.2 M solution of phosphoric acid, and phase B being pure acetonitrile). Flow rate was 1 mL/min, with photodiode-array (PDA) detection (UV at 260, 325 nm, for flavanols and stilbene derivatives at 280 nm), always within 70 min. Best peak separation was achieved using the following combination: 89–75% A (0–35 min); 75–60% A (35–55 min); 60–35% A (55–60 min); and 0–35% A (60–70 min). The concentration of the investigated extract was 50.5 mg/mL and prior to injection, the sample was filtered through PTFE membrane filter. For standards used in the investigation, the concentration were: 0.01 mg/mL for procyanidin B2, 0.15 mg/mL for isoquercetin, 0.26 mg/mL for hyperoside, 0.28 mg/mL for kaempferol-3-O-glucoside, 0.30 mg/mL for neochlorogenic acid and kaempferol, 0.34 mg/mL for gallic and protocatechuic acid, 0.36 mg/mL for quercetin, 0.38 mg/mL for sinapic acid and resveratrol, 0.40 mg/mL for epicatechin and rutin, 0.46 mg/mL for pyrogallol, 0.52 mg/mL for caffeic acid, 0.56 mg/mL for chlorogenic acid, 0.56 mg/mL for ferulic acid, 0.74 mg/mL for p-coumaric acid. The injection volume of standard solutions and also tested extract was 4 µL. 

Identification was based on overlay curves and the retention times. After spectra matching succeeded, results were confirmed by spiking with respective standards to achieve a complete identification using the so-called peak purity test and the peaks that did not fulfill these requirements were not quantified. Quantification was performed by means of external calibration with standards.

#### 2.3.3. GC Analysis of Wild Bilberry Seeds Oil

The oil sample was analyzed after converting the fatty acids to methyl esters, using the AOAC procedure 965.49 [9], with some modifications. A fatty oil sample (45–55 mg) was measured into dry flask, and mixed with a methanol-sulfuric acid mixture, the mixture is carefully heated for 2 h under reflux to enable moderate boiling. After the reaction is complete, the balloon is cooled in a mixture of water and ice. The mixture was washed twice with 50 mL of petroleum ether and once again with 20 mL of distilled water. Afterwards, petrol ether fraction was dried by passing through the anhydrous Na_2_SO_4_. The dried solution is carefully filtered through filter paper to remove the desiccant, transferred to a dry and clean Erlenmeyer flask, and the solvent was evaporated on a vacuum evaporator. The oily residue obtained was weighed, dissolved in methylene chloride, and the samples thus prepared were further analyzed using gas chromatography. The chemical composition of the fatty oil was analyzed using GC and GC/MS technique. GC/MS analyses were performed on a Shimadzu GCMSQP2010 ultra mass spectrometer fitted with a flame ionic detector and coupled with a GC2010 gas chromatograph. The InertCap5 capillary column (60.0 m × 0.25 mm × 0.25 µm) was used for separation. Helium (He), at a split ratio of 1:5 and a linear velocity of 35.2 cm/s was used as a carrier gas. The ion source temperature was 200 °C, injector temperature was 250 °C, detector temperature 300 °C, while column temperature was linearly programmed from 40 to 260 °C (at rate of 4 °C/min), from 260 to 310 (at rate 10 °C/min) and after reaching 310 °C, kept isothermally for 10 min. The derivatized samples were dissolved in the methylene chloride a and consecutively injected always in amount of 1 µL. The content of different compounds was determined on the basis of area of chromatograms and defined as content according to the GC area (the mean of three determinations). The identification of the constituents was performed by comparing their mass spectra and retention indices (RIs) with those obtained from authentic samples and/or listed in the NIST/Wiley mass-spectra libraries, using different types of search (PBM/NIST/AMDIS) and available literature data [10,11]. 

### 2.4. Antioxidant Activity of Wild Bilberry Isolates (Leaves Extract and Seeds Oil)

#### 2.4.1. Radical-Scavenging Activity

In order to perform DPPH assay, method adapted from Brand-Williams et al. [12] was used. Briefly, 300 µL of test solution and 2.7 mL of 0.04 mg/mL methanol DPPH solution were mixed. Test solution was either extract diluted in methanol (extract was diluted in 5 different concentrations: 0.1, 0.25, 0.5, 0.75, and 1.0 mg/mL) or wild bilberry seed oil diluted in methanol (0.25, 0.5, 1.0, 1.5, and 2.0 mg/mL). The absorbance was recorded at 517 nm after 30 min incubation at room temperature in the dark, against methanol as a blank. Free radical scavenging activity was calculated against control solution, containing methanol instead of test solution using the formula:

DPPH radical scavenging capacity (%) = [(A_C_ − A_S_)/A_c_] × 10 (3)

A_S_ was absorption of methanol solution of the extract/oil treated with DPPH radical solution; A_C_ was absorption of control solution.

#### 2.4.2. Ferric-Reducing Antioxidant Power (FRAP)

A slightly modified FRAP assay method by Benzie and Strain [13] was used to determine the antioxidant power of both wild bilberry isolates. To perform FRAP assay, 100 µL of different solutions, previously used in DPPH assay (diluted extract—1.0 mg/mL and diluted seed oil—1.0 mg/mL) and 3.0 mL of freshly prepared FRAP reagent (25 mL of 300 mM acetate buffer pH 3.6 plus 2.5 mL of 10 mM TPTZ solution in 40 mM HCl plus 2.5 mL of 20 mM FeCl_3_ × 6H_2_O) were mixed. The absorbance was recorded at 593 nm against a blank, containing 100 µL of resembling solvent, after 30 min incubation at 37 °C.

The FRAP value was calculated from the calibration curve of FeSO_4_ × 7H_2_O standard solutions, covering the concentration range 100–1000 mmol/L and expressed as mmol Fe^2+^/g extracts. The FRAP values were also estimated for positive controls (BHT and α-tocopherol) in the same manner. All spectrophotometric readings, in both antioxidant activity assays (DPPH and FRAP) were conducted on Evolution 60 UV/Vis Spectrophotometer (Thermo Fisher scientific, Waltham, MA, USA).

### 2.5. Preparation of the Creams with Wild Bilberry Isolates

Samples were prepared by adding heated aqueous phase (75 °C) to the heated oil phase (70 °C) (Table 1) on thermostatic heating plate of magnetic stirrer IKA-MAG (IKA Werke, Staufen, Germany). Propeller rotary laboratory stirrer RW16 basic (IKA Werke, Germany) was used for stirring. At the temperature of 40 °C herbal active substances-wild bilberry leaves extract and wild bilberry seed oil were added into the cream (designated as WB), and the stirring was continued until the preparation had cooled. The placebo cream (sample P) sample did not contain wild bilberry oil or extract.

### 2.6. Skin Study Design

#### Skin Study Design

The testing of the effects of creams was conducted using in vivo non-invasive biophysical techniques. For this purpose, the device Multi Probe Adapter MPA^®^ 9 (Courage & Khazaka Electronic GmbH, Köln, Germany) with different probes, previously calibrated, was used. The probe Corneometer^®^ CM 825 was used for measuring electrical capacitance (EC) which depicts *stratum corneum* hydration, while the probe Tewameter^®^ TM 300 was used as a measuring device for the assessment of the Transepidermal Water Loss (TEWL). The probe Skin-pH-Meter PH 905 was used as a quick tool for measuring the pH of the skin while the erythema index (EI) was measured using Mexameter^®^ MX.

The study lasted for 30 days. The participants who took part in it, were 25 healthy volunteers (mean age 23.36 ± 0.64 years) of both genders (20 women and 5 men), who neither had past or present history of skin diseases, nor used systemic or topical drugs within the last two weeks prior to the study. All volunteers were informed about the study protocol and have signed the written informed consent form. The research was conducted in accordance with the Helsinki Declaration and permitted by the Ethics Committee of the Medical faculty in Niš (Serbia), protocol code 12-6316-2/8 from 16 June 2016. The whole study was carried out in consonance with the guidelines and published recommendations.

The specific areas sized 9 m^2^ on volar part of right forearms of volunteers were determined for treating with cream samples (active—WB and placebo—P) application. In addition, one area was defined as non-treated control (C). All participants were given preparation samples marked with place of application. 

The day before the start of the study, basal values of all biophysical parameters were measured at all designated areas (on volar parts of forearms). The participants were told to apply all the samples on defined areas two times a day, (morning and evening) for 28 days. Also, they were informed not to apply any other product on the test sites during the study. The following measurements were performed after 7, 14, and 28 days of application in the morning, before applying the sample preparations. The last measurement took place 2 days after cessation of application of the samples. Each measurement was conducted under precisely defined conditions to which the volunteers were subjected before the measuring (30 min rest with uncovered forearms, at room temperature 21 ± 2 °C and relative humidity 45 ± 3%).

### 2.7. Examination of Sensory Properties

In order to assess certain sensory characteristics of the investigated cream and emulgel samples, 25 human volunteers were given a questionnaire (Table 2) in which the sensory descriptors were listed. They estimated characteristics of samples before rubbing, during spreading, as well as the feeling after application. All panelists had to choose descriptive term for each sensory attribute listed in questionnaire. The sensory evaluation was carried out in the properly illuminated laboratory, at room temperature 21 ± 2 °C and relative humidity 45 ± 3%.

### 2.8. Statistical Analysis

The results of in vivo measurements were presented as mean ± standard error for each parameter. Measured values were compared to basal values and changes in values during the study were analyzed. Further, samples with herbal active principles were compared to placebo samples and non-treated control. The statistical analysis (Student’s *t*-test and ANOVA) was performed using IBM SPSS Statistics 20.

## 3. Results and Discussion

### 3.1. Chemical Analysis of Wild Bilberry Isolates

#### 3.1.1. Chemical Analysis of Wild Bilberry Leaves Extract

As the phenolic constituents are generally accepted to be active principles of medicinal plants that possess significant biological properties [14], especially when it comes to their antioxidant effects, total phenolic compounds (TP), tannins (TT), flavonoid (TF) content were assessed in wild bilberry leaves extract, as initial screening of its chemical profile. TP content was found to be 217.59 mg GAE/g, TT was 9.17%, while the measured TF content amounted 2.15%. Such findings were in good correlation with the literature data, reiterating leaves of wild bilberry to be the rich source of TP and TF on the one hand, and ethanol to be an adequate solvent for the extraction of stated compounds, on the other [15,16]. 

**Figure 1:**
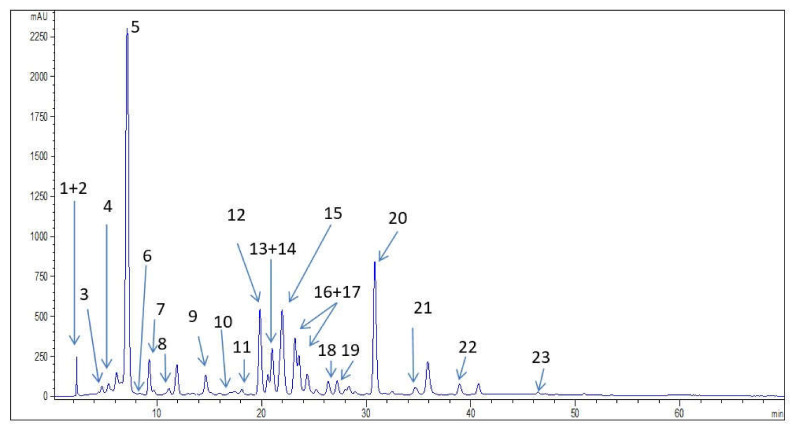
HPLC chromatograms of investigated extract of wild bilberry leaves and phenolic compound identified at 325 nm.

HPLC analysis allowed identification of twenty-three phenolic compounds (Figure 1, Table 3). The presence of phenolic acids in the investigated extract was significant. The predominant phenolic acids were derivatives of hydroxycinnamic acid was led by chlorogenic acid (45.51 mg/g), followed by caffeic acid, protocatechuic, and p-coumaric acid present in the amount ranking from 1 to 3 mg/g. Determined polyphenol acids, present in the investigated extract, but in lower amount, were neochlorogenic, sinapic and ferulic acid. From the group of derivatives of hydroxybenzoic acid, gallic and protocatechuic acid were identified (Table 3). Previous investigation of wild bilberry leaves revealed caffeic acid derivatives to be the most representative group of phenolic compounds [17].

In the case of flavonoids (Figure 1, Table 3) the wild bilberry leaves extract was most abundant in flavones and flavonols, predominantly isoquercetin (14.62 mg/g), followed by rutin (4.73 mg/g), almost equal content of hyperoside and quercetin, and followed by lesser amounts of kaempferol-3-*O*-glucoside and kaempferol (Table 3). The determined content of quercetin in the investigated extract was higher than the results obtained by Hokkannen et al. [18] and Može et al. [4]. Kaempferol was detected in wild bilberry leaves extract which was in accordance with the date obtained by Bljajic et al. [19], while in the work of Hokkanen et al. [18] this compound was not found.

From the group of flavanols, epicatechin was found in the wild bilberry leaves extract which was in accordance with the literature data [18]. Procyanidin was also detected (Figure 1, Table 3). Stilbenes were detected as well in the tested wild bilberry leaves extract (Figure 1, Table 3). The content of resveratrol was 7.25 mg/g, which is to the best of our knowledge the first report of the presence of this compound in the wild bilberry leaves. Pyrogallol was determined to be present in the amount of 2.45 mg/g (Figure 1, Table 3).

#### 3.1.2. Chemical Analysis of Wild Bilberry Seed Oil

GC/MC analysis of wild bilberry seed oil obtained using supercritical CO_2_ extraction (Table 4) confirmed high content of PUFAs, i.e., esters of linoleic (ω-6 fatty acid), α-linolenic (ω-3 fatty acid) and oleic (ω-9 fatty acid) acids. Such results are in agreement with the previously reported findings [1,20]. It has been documented that fatty acids particularly α-linolenic, linoleic, and oleic acids exert beneficial health effects by modulating the signaling pathways regulating inflammatory response, cell differentiation, and proliferation. ω-3 and ω-6 fatty acids play a critical role in normal skin function and appearance [21]. Metabolism of linoleic and α-linolenic acid is limited in the skin. They are considered essential nutrients for the skin, influencing the inflammatory response of the skin. ω-6 fatty acids play an important role in the structural integrity and barrier function of the skin, while topical application of certain ω-3 fatty acids lessens UV-induced photo damage, signs of skin aging and inflammatory skin responses [22,23]. Oils abundant in oleic acid are richer and heavier in consistency. They are extra-occlusive and seal in moisture really well. Oleic acid is absorbed well by the skin, has anti-inflammatory and skin softening properties [24].

### 3.2. Antioxidant Activity of Wild Bilberry Isolates

To evaluate the antioxidant properties, two different assays were employed to avoid any possible incorrectness in the interpretation of the obtained results regarding the antioxidant properties of the investigated extracts, considering the differences in their working principles. The antioxidant potential of all extracts was evaluated using the DPPH and FRAP tests. The DPPH assay is based on the hydrogen donating capacity to scavenge DPPH radicals, while the FRAP assay is an electron transfer-based test measuring the substance ability to reduce Fe^3+^ to Fe^2+^.

IC_50_ values of the wild bilberry leaves extract was 2.13 mg/mL (Table 5). The activity of the investigated extract was very close to positive control, BHT (IC_50_ = 2.07 mg/mL), meaning that it possessed high antioxidant capacity. However, antioxidant activity of the tested extract was lower compared to α-tocopherol (Table 5). In comparison to early published data [25], measured IC_50_ value of the extract was lower indicating its higher the hydrogen donating capacity.

FRAP assay (Table 5) indicated much lower reducing power (lower FRAP value) of the investigated extract compared to the positive controls and somewhat lower value when compared to the results cited in the literature [4].

Antioxidant capacity of plant extracts are generally connected to the presence of hydroxyl groups in the structure of the phenolic compounds that might contribute to the stated activity [26,27]. Also, antioxidant effects might depend on the structure and interaction between extracted phenolic compounds [28]. When it comes to the antioxidant activity of phenolic acids, it depends on the number and positions of the hydroxyl groups in relation to the carboxyl functional group; meaning it increases with increasing degree of hydroxylation. For instance, trihydroxylated gallic acid, detected in the investigated extract, was previously shown to possess a high level of antioxidant capacity [26]. Similarly, chlorogenic acid, which was the most abundant phenolic acid in the tested extract, was reported to mitigate oxidative stress, and hence the related adverse effects associated with an unbalanced intracellular redox state [29].

The identified flavonoids derivatives in the investigated extract, quercetin and kaempferol might provide extremely active free-radical scavenger agents, given that they possess the catechol moiety with double bonds at C2–C3 and 3-OH [26]. Resveratrol, found in the investigated extract, was previously found to possess the strong ability to capture free radicals and this property was related to the presence of three hydroxyl groups, as well as the presence of aromatic rings and a double bond in the molecule [30]. Also, pyrogallol, identified in the investigated extract, was reported to exhibit high antioxidant potential, due to the high molecular weight and high degree of hydroxylation of aromatic ring [31].

Wild bilberry seed oil demonstrated lower antioxidant activity compared to the wild bilberry leaves extract in both in vitro assays (Table 5). In DPPH test, obtained IC_50_ for the investigated oil (3.37 mg/mL) was somewhat higher compared to the tested extract (IC_50_ = 2.13 mg/mL), and BHT (IC_50_ = 2.07 mg/mL) indicating good antioxidative capacity. As in the case of wild bilberry extract, IC_50_ value of the oil was quite higher in comparison to α-tocopherol (IC_50_ = 0.47 mg/mL). On the other hand, FRAP value of tested oil (0.2045 mmol Fe^2+^/g of extract) was very low compared to the investigated extract (3.6348 mmol Fe^2+^/g of extract), and particularly in comparison to the positive controls (11.3152 and 16.9616 mmol Fe^2+^/g of extract for BHT and α-tocopherol, respectively). Nevertheless, detected antioxidant activity of wild bilberry oil assessed using DPPH and FRAP test were higher compared to the data presented in literature [1]. It is known that wild bilberry seed oil is a rich source of essential fatty acids with favorable ω6/ω3 ratio (≤1) and high content of PUFAs [21], as confirmed in the current study (Table 4). On the other hand, wild bilberry seed oil has lower content of antioxidants such as vitamin E and carotenoids compared to other berry seed oils [32]. 

### 3.3. In Vivo Investigations of Cream with Wild Bilberry Isolates

#### Skin Effects of the Investigated Cream Assessed via Biophysical Measurements

**Figure 2:**
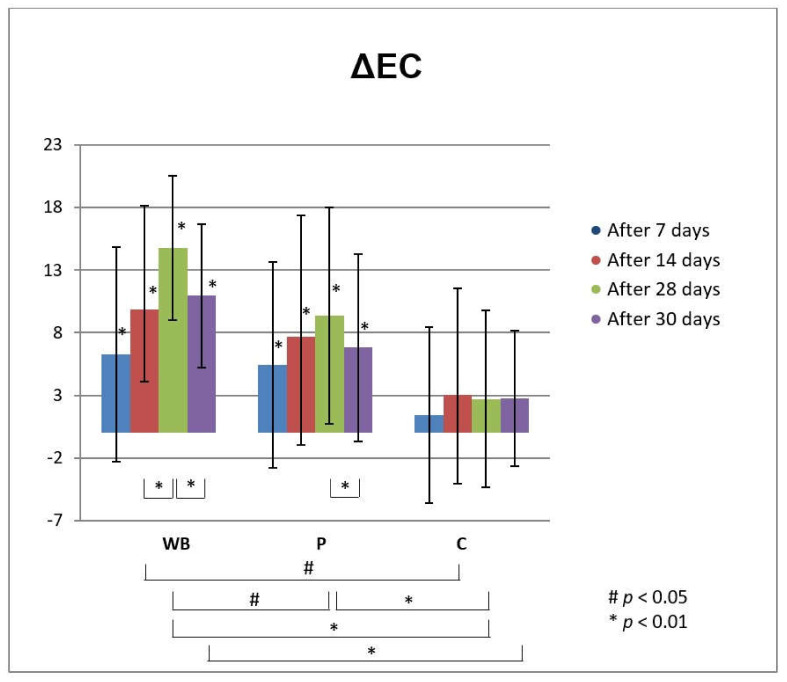
In vivo determined absolute changes relative to basal values of electrical capacitance (EC) for investigated creams (active cream—WB, placebo cream—P) after 7, 14, 28 days of application and 2 days after cessation of application, compared to untreated control (C). Significant differences are being marked with # (*p* < 0.01) and * (*p* < 0.05).

The increase of electrical capacitance (EC) after application of both investigated creams (WB and P) indicated their moisturizing effect on skin (Figure 2). Namely, cream with wild bilberry isolates-leaves extract and seeds oil (sample WB) significantly increased the hydration of *stratum corneum* in all assessed time points, compared to the initially measured values. Starting from the measurement at day 14, this increase was also significant compared to C test-site, where none of the tested creams was applied. Moreover, the increase in *stratum corneum* hydration after treatment with this sample between 14th and 28th day was also statistically significant. On the other hand, the use of placebo cream (P) led to an increase in *stratum corneum* hydration which was statistically significant compared to the initially measured values (before treatment) but not to the NC. The exception was the measurement after the 28 days of treatment with this sample, which was significant in relation to the C, but also in relation to the cream WB. Based on such findings, it may be speculated that the observed significant moisturizing skin effect of cream with wild bilberry isolates is related to the presence of active compounds, and not the vehicle itself. Two days after cessation of use of both tested creams (day 30), there was a significant decrease in hydration compared to 28th day of the measurement. However, this value was still significantly higher than the initially measured one, and in the case of sample WB also in comparison to non-treated control test-site (C).

Dry, inflexible and damaged skin is not just an aesthetic problem, while *stratum corneum* needs appropriate amount of water in order to fulfill its barrier function, i.e., proliferation and differentiation of the epidermal cells cannot be well regulated without adequate *stratum corneum* hydration. Therefore, moisturizing topical preparations have shown positive effect on the skin, especially dry skin [33]. In our study, it was demonstrated that wild bilberry leaves extract and seed oil may influence moisture of the skin. Previously described HPLC analysis of the wild bilberry leaves extract used in our study revealed that it was rich in chlorogenic acid (45.51%), and isoquercetin (14.62%), shown to have some beneficial skin effects. For instance, in addition to being a well-known antioxidant, chlorogenic acid has a role in skin cancer prevention and skin-photo protection [34]. Further, isoquercitrin from herbal extract revealed a dose-dependent inhibitory action against edema induced by allergic contact dermatitis, in addition to other phenolic compounds including gallic acid, quercetin, and kaempferol, also present in the wild bilberry leaves extract used in this study. Isoquercitrin, isolated from acetone extract of persimmon fruit peel, was also found to be a potent inhibitor of melanin production by suppressing tyrosinase expression in mouse B16 melanoma cells [35]. Also, in the study investigating wound-healing effects of cream with isoquercetin, there was a significant increase in the percentage of wound contraction and a significant decrease in the period of epithelialization in isoquercetin-based cream-treated groups as compared with the control group. Histological analysis revealed isoquercetin 0.06% *w*/*w* cream treatment resulted in almost complete re-epithelialization and re-structuring of the wound tissue, alongside a significant rise in thiobarbituric acid reactive substances (TBARS) and a decrease in glutathione (GSH) levels in the burn injury group which was reversed to a major extent by the application of isoquercetin-based cream [36]. In addition to chemical constituents of wild bilberry leaves extract present in the water phase of the tested cream, components from the oil phase as well (originating from the wild bilberry seed oil) might have contributed to its observed moisturizing effects. Namely, it has been shown that after application of emulsions on the skin, evaporation of the greatest percentage of water, known as the evaporation phase, is followed by the phase in which lipids from the emulsion penetrate into the epidermis and lead to increased skin hydration (lipidization phase) [37]. In this connection, previous studies reported linoleic acid and α-linolenic acid, which were found in great amount in the investigated wild bilberry seed oil used in the tested creams, to be safe adjunctive treatments for many skin disorders, including atopic dermatitis, psoriasis, acne vulgaris, systemic lupus erythematosus, nonmelanoma skin cancer, and melanoma [38]. In addition, a recent patent described the invention related to the usage of the *cis*-9, trans-11 isomer of conjugated linoleic acid or a salt or ester thereof to treat or prevent skin inflammation [39].

**Figure 3:**
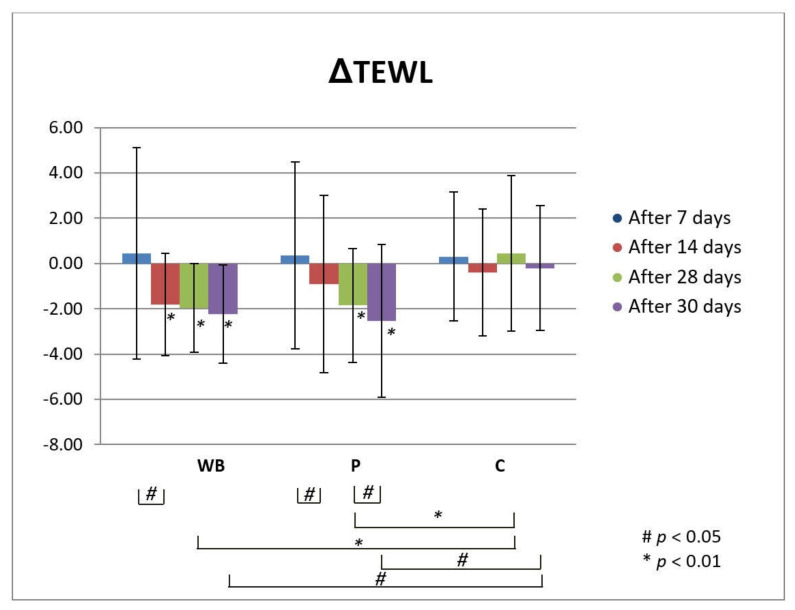
In vivo determined absolute changes relative to basal values of TEWL for investigated creams (active cream—WB, placebo cream—P) after 7, 14, 28 days of application and 2 days after cessation of application, compared to untreated control (C). Significant differences are being marked with # (*p* < 0.01) and * (*p* < 0.05).

TEWL is a parameter that indicates the permeability and integrity of the skin. Therefore, low TEWL values are linked to healthy and normal skin, while increased values imply damaged skin [40,41,42,43]. Application of both investigated creams (WB and P) led to the significant decrease of TEWL i.e., to the improvement of the skin barrier, compared to the initially assessed values at all tested time points (Figure 3). Also, the observed decrease of TEWL was significant between 7th and 14th day of application of both creams with and without the wild bilberry isolates (WB and P, respectively). Also, on 28th day of the experiment, TEWL decrease after usage of both creams was significant in comparison to the non-treated control site (C). It should be emphasized that even two days after discontinuation of the treatment with both investigated creams (day 30 of the experiment), further decrease of TEWL was observed, which was significant compared to the initially assessed values and non-treated control (C) in the case of both samples (WB and P), and also compared to the last day of application in the case of placebo sample (day 28 of the experiment). However, there was no significant difference in the changes of TEWL between the tested creams at no time point of the experiment. Such finding indicated increase of TEWL to be predominantly attributed to the vehicle [37], rather that active substances originating from wild bilberry isolates.

**Figure 4:**
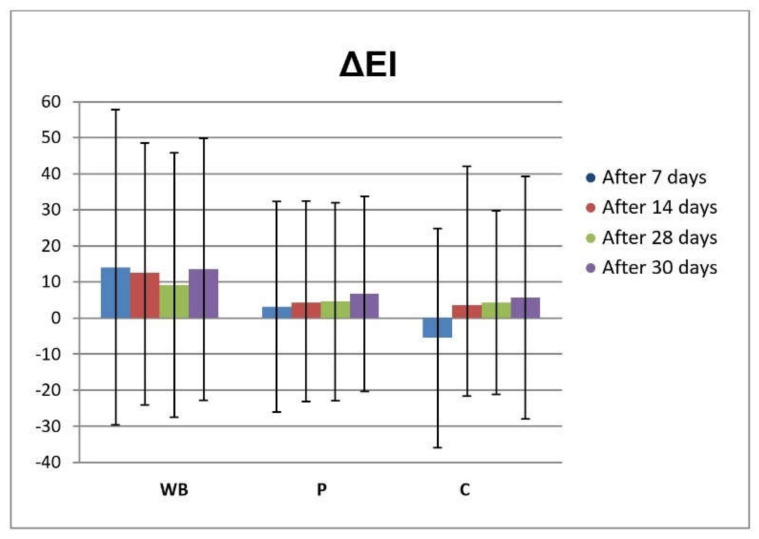
In vivo determined absolute changes relative to basal values of EI for investigated creams (active cream—WB, placebo cream—P) after 7, 14, 28 days of application and 2 days after cessation of application, compared to untreated control (C).

Another good indicator of skin barrier function is EI. Erythema occurs after the skin was injured or exposed to irritants [44]. During the in vivo study, there were no statistically significant changes in EI in assessed time points after application of the tested cream samples (WB and P), compared to the neither basal values nor non-treated control test-site (C), suggesting good tolerability on the skin (Figure 4).

**Figure 5:**
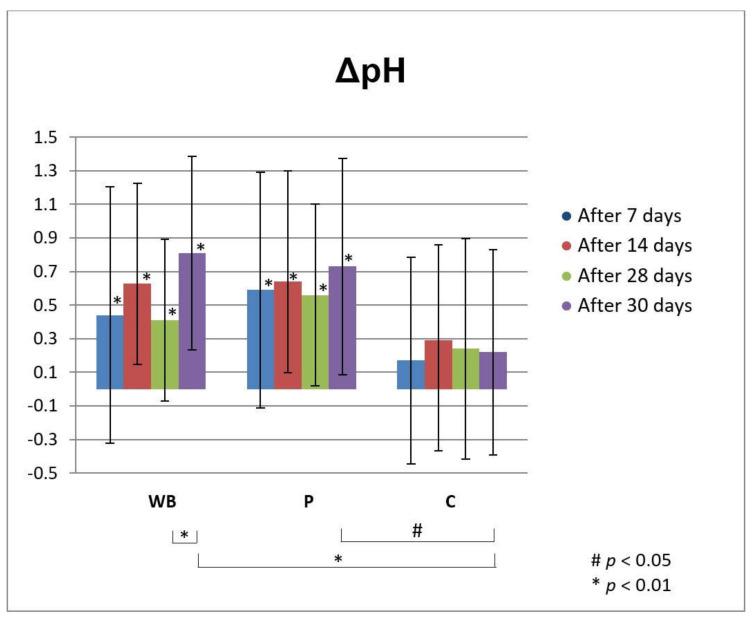
In vivo determined absolute changes relative to basal values of pH for investigated creams (active cream—WB, placebo cream—P) after 7, 14, 28 days of application and 2 days after cessation of application (blue, red, green and purple bar, respectively), compared to untreated control (C). Significant differences are being marked with # (*p* < 0.01) and * (*p* < 0.05).

From the 7th day of measurement, the increase in skin pH after treatment with both tested creams was statistically significant compared to the initially measured values. In comparison with the non-treated control test-site (C), pH changes were statistically significant only on the 30th day after the initial measurement (2nd day after discontinuation of treatment) for both tested samples (Figure 5). Also, the increase of pH value two days after discontinuation of treatment with WB was significant compared to day 28 of the experiment. However, the measured pH values were in the range of normal skin pH values (4–6) during the whole experiment. It is very important to have preserved skin pH value after application of topical preparations, while pH can influence the skin barrier function, thus its change can disrupt the antibacterial protection and homeostasis of the skin [45].

As seen in Table 6, both samples revealed good application properties, which are very important from the user’s point of view. They were characterized as semisolid, easy to spread, not sticky, and not greasy by panelists. Also, results of the organoleptic evaluation of both creams showed that they were described as slightly shiny and creams with fast absorption rate. After application, moderate, slightly sticky film was noticed by majority of panelists and their skin was slightly shiny, but they declared that their skin was not greasy. 

## 4. Conclusions

Traditionally, bilberry leaves were used for the treatment of various health impairments as the consequence of oxidative stress, some of them causing the skin diseases. Although bilberry fruits have been studied as the valuable source of anthocyanins and proanthocyanins, the research focusing on the seed oil fatty composition is scarce. In addition, bilberry seeds and leaves represent the byproduct in food industry. In our work we used the bilberry leaves extract and seed oil to formulate preparation for skin application with the aim to bring the connection between their antioxidant potential, the chemical profile of the abovementioned bioactives, and their beneficial effects on the skin. The presence of chlorogenic acid, isoquercetin, and resveratrol as most abundant phenolic compounds in bilberry leaves extract, and α-linolenic, linoleic, and oleic acids (with the stress on existing favorable ratio of 0.98 for α-linolenic (ω-3), and linoleic (ω-6) acids) in bilberry seeds oil might be responsible for the obtained result, i.e., satisfactory antioxidant potential of both investigated bilberry isolates assessed via two in vitro assays (DPPH and FRAP), yet with leaves extract revealing stronger antioxidant capacity. O/W cream with the investigated wild bilberry isolates as active components revealed the potential to significantly increase the hydration of *stratum corneum* while concomitantly improving the skin barrier function and showing good tolerability and preserved pH value of the skin after application. Adding to these good sensory properties of the formulated cream containing wild bilberry isolates as active principles, such preparation might be potentially used in the skin impairments accompanied by oxidative stress and/or dry skin.

## Figures and Tables

**Table 1:** Qualitative and quantitative composition (%, (*w*/*w*)) of cream samples: active sample (WB) and placebo (P).

Component		WB	P	Function
Trade Name	INCI Name			
**Oil phase**				
Myritol^®^ 318	Caprylic/capric triglycerides	5.50	5.50	emollient
Sabowax^®^ AE	Glyceryl Stearate (and) ceteareth-20 (and) ceteareth-12 (and) cetearyl alcohol (and) cetyl palmitate	8.50	8.50	O/W emulsifier, surfactant
Lanette 16	Cetyl alcohol	0.75	0.75	emollient
Stearyl alcohol	Stearyl alcohol	0.75	0.75	emollient
**Water phase**				
Glycerin	Glycerin	2.00	2.00	humectant
Sodium benzoate	Sodium benzoate	0.50	0.50	preservative
Purified water	Water distilled to	100.0	100.0	water phase
**Active substances of plant origin**				
Wild bilberry oil	*Vaccinium myrtillus* L., seed oil	6.00	-	active component
Wild bilberry extract	*Vaccinium myrtillus* L. leaves maceration with 70% (*v*/*v*) ethanol	6.00	-	active component

**Table 2:** Sensory evaluation questionnaire.

Before Application	
**Consistency**	liquid/semi-solid
**Gloss level**	matt/pearl gloss/slightly gloss/gloss/very gloss
**During application**	
**Spreadability**	easy to spread/difficult to spread/very difficult to spread
**Adhesion**	not sticky/slightly sticky/sticky/very sticky
**Density**	rare/slightly dense/dense/very dense
**Grease**	not greasy/slightly greasy/greasy/very greasy
**Gloss**	not shiny/slightly shiny/ shiny/very shiny
**Absorption rate**	slow/moderate/fast
**After application**	
**Residual film**	no film/moderate film/expressive film
**Stickiness**	not sticky/slightly sticky/sticky/very sticky
**Grease**	not greasy/slightly greasy/greasy/very greasy
**Gloss**	not shiny/slightly shiny/shiny/very shiny

**Table 3:** Content of phenolic compounds determined by HPLC in the investigated wild bilberry leaves extract [mg/g].

Phenolic Compounds	Numbers in Figure 1	Wild Bilberry Leaves Extract
**Derivatives of hydroxycinnamic acid**		
Neochlorogenic acid	3	0.34
Chlorogenic acid	5	45.51
Chlorogenic acid derivative	21	*
Caffeic acid	7	1.95
p-coumaric acid	9	1.26
Sinapic acid	10	0.18
Ferulic acid	11	0.26
**Derivatives of hydroxybenzoic acid**		
Gallic acid	1	0.80
Protocatechuic acid	4	1.40
**Flavones and flavonols**		
Rutin	13	4.73
Hyperoside	14	2.51
Isoquercetin	15	14.62
Kaempferol-3-*O*-glucoside	18	1.56
Quercetin	22	2.11
Quercetin derivative 1	19	*
Kaempferol	23	0.10
**Flavanols**		
Procyanidin B2	6	1.29
Epicatechin	8	7.53
**Stilbenes**		
Resveratrol	20	7.25
Stilbenoid derivative 1	12	*
Stilbenoid derivative 2	16	*
Stilbenoid derivative 3	17	*
**Pyrogallol**	2	2.45

* tentative identification.

**Table 4:** Content of compounds in wild bilberry seeds oil determined by GC-MC analysis.

Name	CAS	RI—Retention Index	%
Methyl hexadecanoate	112-39-0	1921	4.61
Methyl linoleate, ω-6	112-63-0	2095	38.68
Methyl oleate, ω-9	112-62-9	2108	18.14
Methyl α-linolenate, ω-3	301-00-8	2113	37.79
Methyl stearate (methyl octadecanoate)	112-61-8	2124	0.55
8,11,14-eicosatrienoic acid, methyl ester	17364-32-8	2249	0.11
*cis*-11,14-eicosadienoic acid, methyl ester	24603-02-7	2305	0.11

**Table 5:** Antioxidative activity of the investigated samples: wild bilberry isolates (leaves extract and seed oil) and positive controls (α-tocopherol and BHT).

Samples	DPPH Assay (IC_50_ (mg/mL))	FRAP Assay (mmol Fe^2+^/g of Extract)
**Wild bilberry leaves extract**	2.13	3.6348
**Wild bilberry seed oil**	3.37	0.2045
**α-tocopherol**	0.47	16.9616
**BHT**	2.07	11.3152

**Table 6:** The results of sensory analysis of the investigated samples *.

Before Application						
	**Consistency**	**Gloss Level**				
**WB**	Semisolid (100.0%)	Matt (69.2%)				
**P**	Semisolid (92.3%)	Slightly gloss (46.2%)				
**During Application**						
	**Spreadability**	**Adhesion**	**Density**	**Grease**	**Gloss**	**Absorption Rate**
**WB**	Easy to spread (61.5%)	Not sticky (61.5%)	Slightly dense (46.2%)	Not greasy (46.2%)	Slightly shiny (69.2%)	Fast (61.5%)
**P**	Easy to spread (76.9%)	Not sticky (46.2%)	Dense (46.2%)	Not greasy (53.8%)	Slightly shiny (53.8%)	Fast (50%)
**After Application**						
	**Residual Film**	**Stickiness**	**Grease**	**Gloss**		
**WB**	Moderate film (69.2%)	Slightly sticky (61.5%)	Not greasy (53.8%)	Slightly shiny (100%)		
**P**	Moderate film (53.8%)	Slightly sticky (46.2%)	Not greasy (69.2%)	Slightly shiny (46.2%)		

* The table shows the most common answers with the percentage of respondents who rated the creams (active cream—WB, placebo cream—P) in this manner.

## Data Availability

The additional data regarding the performed investigations are available by request from authors.
